# Lepra Bubalorum, a Potential Reservoir of *Mycobacterium leprae*

**DOI:** 10.3389/fmicb.2021.786921

**Published:** 2021-12-02

**Authors:** William R. Faber, Henk Menke, Victor Rutten, Toine Pieters

**Affiliations:** ^1^Department of Dermatology, Amsterdam University Medical Centre (UMC), University of Amsterdam, Amsterdam, Netherlands; ^2^Faculty of Science, Freudenthal Institute, Utrecht Institute for Pharmaceutical Sciences (UIPS), Utrecht University, Utrecht, Netherlands; ^3^Division of Infectious Disease and Immunology, Department of Biomolecular Health Sciences, Faculty of Veterinary Medicine, Utrecht University, Utrecht, Netherlands; ^4^Department of Veterinary Tropical Diseases, Faculty of Veterinary Science, University of Pretoria, Pretoria, South Africa

**Keywords:** *M. leprae*, *M. leprae* complex, lepra bubalorum, Indonesia, animal reservoir, water buffaloes

## Abstract

In 1926, a mycobacterial skin disease was observed in water buffaloes by researchers in Indonesia. The disease was designated as skin tuberculosis, though it was hypothesized that it might be a form of leprosy or a leprosy-like disease. In a follow-up study (Ph.D. thesis Lobel, 1934, Utrecht University, Netherlands) a similar nodular skin disease was described in Indonesian water buffaloes and named “lepra bubalorum” or “nodular leprosy.” Two decades later Kraneveld and Roza (1954) reported that, so far, the diagnosis lepra bubalorum had been made in 146 cases in Indonesia. After a final series of research reports by Indonesian veterinarians in 1961, no subsequent cases were published. Based on information from these reports, it can be concluded that, even though evidence of nerve involvement in buffaloes was not reported, similarities exist between lepra bubalorum and Hansen’s disease (leprosy), i.e., nodular skin lesions with a chronic course and microscopically granulomatous reactions with AFB in globi in vacuoles. This raises the question as to whether these historical cases might indeed have been caused by *Mycobacterium leprae*, *Mycobacterium lepromatosis* or another representative of the *M. leprae* complex. The future use of state-of-the-art molecular techniques may answer this question and may also help to answer the question whether water buffaloes should be considered as a potential natural reservoir of the causative pathogen of Hansen’s disease.

## Introduction

Leprosy, also known as Hansen’s disease, results from infection with *Mycobacterium leprae* (*M. leprae*) or *Mycobacterium lepromatosis* (*M. lepromatosis*). Although originally it was assumed that only humans could be affected, during the last decades evidence has mounted that other species may be affected as well.

Until now, three non-human wild-life reservoirs of *M. leprae* have been reported: armadillos in the Americas (Sharma et al., 2015), red squirrels on the British Isles (Avanzi et al., 2016) and various non-human primates in Africa and Asia (Honap et al., 2018; Hockings et al., 2021). *M. lepromatosis* has also been detected in the British red squirrels (Simpson et al., 2015). Humans and armadillos in the Southern United States share a specific *M. leprae* strain (SNP subtype 3I-2-v1) (Sharma et al., 2015). This finding is highly suggestive of a zoonotic and/or an anthroponotic transmission pathway between humans and armadillos. Human exposure to armadillos may eventually lead to leprosy or an indication of infection as evidenced by increased anti-PGL I antibody levels (Penna et al., 2016). Exposure to an animal or to its excreta is influenced by human behaviour. For example, activities such as hunting and/or preparing armadillos as food can be expected to cause different risks of transmission, depending on exposure intensity and frequency (Silva da et al., 2018). It is unclear whether armadillo transmission risks are confounded by environmental exposure to *M. leprae*, caused by shedding of leprosy bacteria by infected armadillos. Presence of *M. leprae* RNA in environmental samples indicated the presence of viable microorganisms in soil and water samples in Brazil and India (Mohanty et al., 2016; Turankar et al., 2016; Arraes et al., 2017; Holanda et al., 2017; Tió-Coma et al., 2019). In addition, amoebae were found to be capable of taking up *M. leprae* by phagocytosis. Inside amoebae cysts *M. leprae* remained viable up to 8 months (Wheat et al., 2014). This mechanism might contribute to environmental survival in the absence of a mammalian host.

Since the incidence of leprosy has declined slowly in certain areas, it is of interest to investigate whether more reservoirs exist from which transmission of causative pathogens is possible. In this article based on a series of historical case studies in Indonesia we hypothesize that a mycobacterial infection in water buffaloes called Lepra Bubalorum might be connected to *M. leprae*.

## Lepra Bubalorum, Historical Cases in Indonesia

In 1926, a mycobacteriosis called skin-tuberculosis was reported in Indonesian water buffaloes (Kok and Roesli, 1926). The report concerned three water buffaloes from the Semarang area in Middle Java, Indonesia, which had lesions on different body areas ranging from marble to potato size, to even bigger swellings with irregular borders. The lesions were confined to the skin and in one animal the right bow gland was affected. The consistency of the lesions ranged from solid to caseous degeneration with calcification, to lesions with fluctuating consistency and purulent fluid. The animals’ general health was not affected. Ziehl-Neelsen staining of smears from lesions showed acid fast bacilli (AFB) with a tendency to cluster, which contrasts with tubercle bacilli. Granular staining pattern was also seen. The injection of suspensions derived from early (solid) lesions and from pus into guinea pigs did not result in the development of lesions nor was inoculation on different solid culture media successful. The authors concluded that the diagnosis skin tuberculosis could not be established and that it might instead be a form of leprosy, since this disease was rather prominent in indigenous human populations, as was close contact between the owners and their water buffaloes.

**FIGURE 1:**
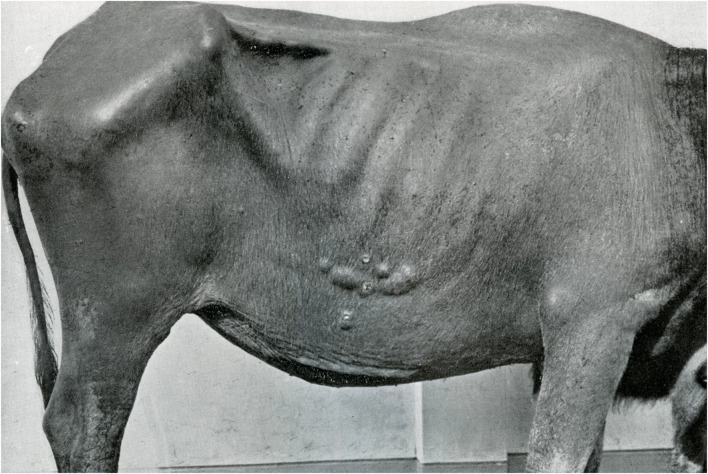
Lesions in water buffalo confined to one skin area.

In 1934, the veterinary surgeon Lobel defended his Ph.D. thesis entitled “Lepra Bubalorum” at Utrecht University, Netherlands (Lobel, 1934) and also published his findings in the International Journal of Leprosy (Lobel, 1936). His research concerned eighteen water buffaloes with “Lepra Bubalorum” from 4 districts in Java and one in Celebes (nowadays Sulawesi). The most common manifestations observed were skin nodules, generally measuring 0.5–6 cm in size and sometimes clustered in large conglomerates (Figure 1). The consistency was firm, although sometimes with fluctuation in large lesions. Ulceration of the nodules was observed, with flattening of the lesions back to skin level in course of a few years. Depigmentation and hair loss might occur. The extent of the skin lesions varied greatly, but was in general limited to one skin area. In two of the animals, ulceration and granulation of the nasal mucosa was also seen.

**FIGURE 2:**
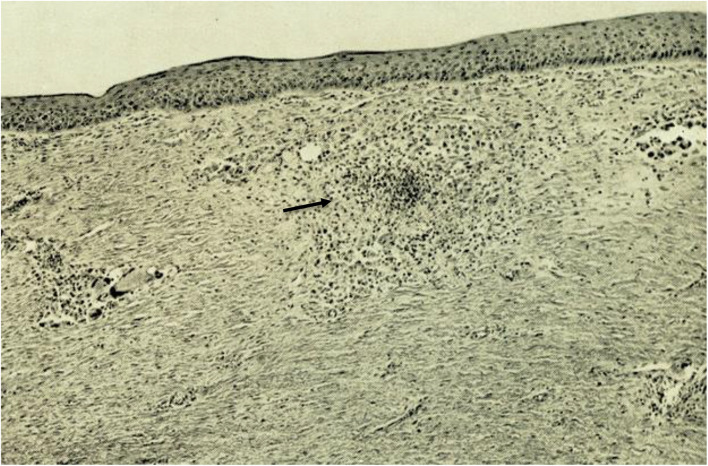
Granulomatous reaction in the dermis; see arrow.

**FIGURE 3:**
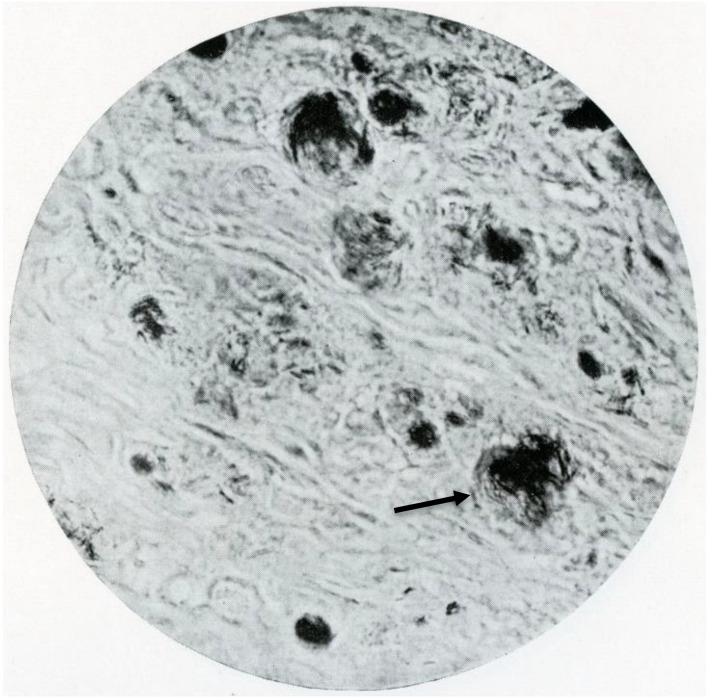
Vacuoles with AFB in globi and granular forms; see arrow.

Macroscopically, the nodules were mostly well defined and localised in the cutaneous layer. Microscopically, granulomas could be identified which were, as a rule, localised in the dermis with a small sub-epidermal free zone (Figure 2). The cellular constituents were macrophages, giant cells including Langhans’ type and fibroblasts. Lymphoid cells were less numerous. Necrosis was present in irregular small areas. The cytoplasm of the macrophages had a foamy appearance with vacuoles, in general one per cell. Large vacuole-like cavities were also localized extracellularly. The vacuoles and the vacuole-like cavities contained clusters, bundles, or globi of AFB as well as granular forms (Figure 3). The bacilli were not found in endothelial cells nor in nerves.

Clinically, Lobel (1934) compared this disease to “lepra tuberosa” in man, based on the similarity of the skin manifestations and the chronic, prolonged course of the disease.

In water buffaloes the disease was confined to the skin whereas in lepra tuberosa nerves and internal organs are also involved. Ulcerations and mutilations—tropho-neurotic in origin in human leprosy—were not observed.

In two animals with extensive skin lesions an acute episode was seen, with a rise in temperature and generalised erythema of the skin with hyperesthesia. A few days later, many small elevated hyperaemic spots developed which disappeared after 10–24 days. These acute skin manifestations with a febrile period may resemble type II reactions or erythema nodosum leprosum (ENL) in leprosy in humans.

However, so-called Virchow cells in human leprosy generally contain several vacuoles, whereas in the buffaloes only one vacuole is seen.

*In vivo* inoculation experiments in buffaloes, guinea pigs, rabbits, chickens, laboratory rats and mice, using various inoculation routes (contact, intracutaneous, subcutaneous, intramuscular, intraperitoneal, and intravenous) did not result in disease signs, with observation periods of 7 months up to 2 years. The material used for inoculation were homogenised skin nodules in which numerous intact rods of bacteria were present. These negative results of bacterial cultures and animal inoculations suggest a close relationship between the AFB in the lesions of buffaloes and *M. leprae*. In conclusion, Lobel stated that this disease in water buffaloes should be named “lepra bubalorum” or “nodular leprosy.”

Clinical manifestations similar to those in water buffaloes have also been found in cattle in Indonesia, as first reported by Meyer and Walandouw (1935). In these animals nodules were present on the skin of all body regions.

In 1940 a case of lepra bubalorum with a different clinical expression, namely elevated tumor-like lesions, was described (Kraneveld and Lobel, 1940).

In a review article by Kraneveld and Roza (1954) entitled “lepra bubalorum and lepra bovina in Indonesia,” the authors mention that Lobel (1935) presented data on 63 cases of lepra bubalorum (37 of which were from Palu district in Sulawesi). By 1940, 138 cases had been registered of which 80 from Palu district. Unfortunately all manuscript data were lost during World War II.

The Indonesian veterinarians Ressang and Sutarjo (1961) also published results of experimental transmission of material from water buffalo lesions into a number of animals: guinea pig, mouse, white rat, rabbit, calf, heifer, horse, sheep, goat, dog, cat, monkey, and alligator. The material was obtained from a fresh granuloma nodule and administered as aqueous and oily emulsions. Several routes of application were used: intracutaneous, subcutaneous, intramuscular, intratesticular, skin punctures, skin scarification, and brushing on unabraded skin. Some of the small animals were pre-treated with total body irradiation or cortisone to suppress the immune system. The animals were observed for a period of 10–25 months. Only in one animal, a heifer, growth of the inoculum was seen, from 5 mm to 6 cm after 7 months; a second passage resulted in a nodule of 3 cm which histopathologically showed vacuolisation of granuloma cells, and only granular forms of the AFB were seen.

**FIGURE 4:**
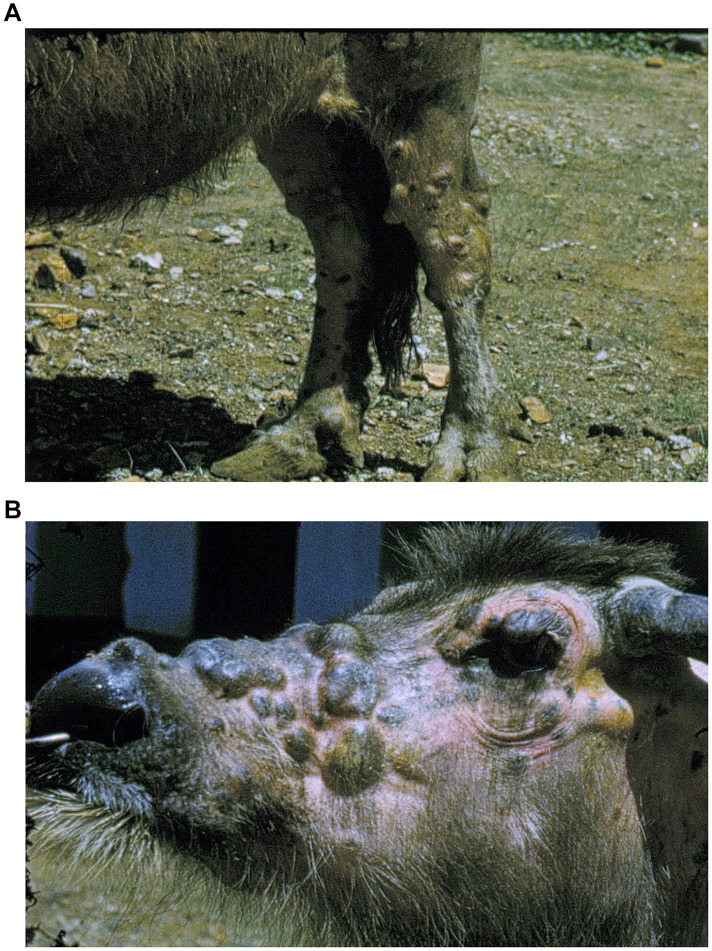
**(A)** Water buffalo with extensive skin lesions. **(B)** Image from colour film by Ressang (1961).

Ressang (1961) described one buffalo cow with nodules over its whole body (Figures 4A,B). He performed histopathological examination of a nerve trunk embedded in granulomatous tissue; the nerve bundles were unaffected and no bacterial growth in the perineurium was seen. Since this last report, no further cases have been published. In 1996 the Indonesian pathologist Budiarso (1996) wrote a resume of what was known of this disease at that time entitled “Is there any relationship between water buffalo leprosy, which has only been found in Indonesia, and human leprosy in Sulawesi?.” In total around 200 cases of lepra bubalorum have been recorded in buffaloes, of which 80% in Sulawesi. Clinically and histopathologically it resembles lepromatous leprosy in humans. Budiarso raises the question whether lepra bubalorum is an anthropozoonosis, and advocates to integrate veterinary and medical knowledge in the study of leprosy in Sulawesi. This is exemplary for a One Health approach.

Since the majority of cases at that time were found in Palu district in Sulawesi, in 2020 we tried to find out whether it still occurs in that area. With the support of Prof M Hatta, Hasanuddin University, Makassar (Sulawesi), we obtained information (personal communication) from microbiologists, primary health care workers, and villagers. None were aware of any case resembling lepra bubalorum at that time (personal communication).

## Lepra Bubalorum a Reservoir of *Mycobacterium Leprae* in Past and Present?

In line with Budiarso (1996) we hypothesize that lepra bubalorum may have been a natural reservoir and an additional example of an animal host of *M. leprae*. Clinically evident similarities between human leprosy and lepra bubalorum exist. Likewise, episodes resembling type II reactions or (ENL) in human leprosy occur in buffaloes, but microscopic investigation to confirm this, were not performed. AFB are as a rule found intracellularly but in contrast to human leprosy, neither in nerves nor in endothelial cells, and there is no evidence of clinical nerve involvement. As in human leprosy, culture of the mycobacteria on an artificial medium and inoculation experiments in a variety of animals failed.

Leprosy (both in the past and at present) is common in Indonesia. It is one of the three countries (together with India and Brazil) which reported more than 10,000 new cases in 2020 (World Health Organization, 2021). Since 1961, no further cases of lepra bubalorum have been reported and one can only speculate why this is the case. The prevalence of infectious diseases may change rather unexpectedly. For example an outbreak of Buruli Ulcer (BU), another mycobacterial disease, named after Buruli county in Uganda was linked to unprecedented flooding in that area (Barker, 1971). The epidemiology of BU is linked to environmental factors such as rural areas near wetlands and swamps and slow-moving rivers as well as environmental disturbances like deforestations, dam construction and agriculture (Merritt et al., 2010). BU is not diagnosed any more in Uganda in present times. In Suriname so-called mycobacterial ulcers were seen in the last century and one case of BU from Surinam has been published (Faber et al., 2015). BU is still present in the neighboring country of French Guyana, where the decline could have been caused by improving living conditions, access to health care and prophylactic recommendations (Douine et al., 2017).

Recently feline leprosy has been described to be caused by a variety of mycobacteria of which *Candidatus* “Mycobacterium lepraefelis” is closely related to *M. leprae* and *M. lepromatosis* (O’Brien et al., 2017). It is also speculated that *M. leprae*, *M. lepromatosis*, *Candidatus* “M. lepraefelis,” *Candidatus* “M visible,” and the cattle organism that causes nodular thelitis (Pin et al., 2014) will 1 day be grouped together as the *M. leprae* complex (Ploemacher et al., 2020). It is therefore also possible that lepra bubalorum is not caused by *M. leprae* or *M. lepromatosis* but by another (as yet unknown) mycobacterium within or beyond the *M. leprae* complex. To precisely define the mycobacterial cause of lepra bubalorum, molecular techniques are in place to readily identify the genome, as well as its relationship with *M. leprae* or *M. lepromatosis* or its membership of the *M. leprae* complex (Ploemacher et al., 2020).

In a recent review on reservoirs and transmission of leprosy (Malik and O’Brien, 2017; Ploemacher et al., 2020) point out that a One Health approach is warranted to explore whether other reservoirs with the potential for transmission exist. In this regard, further research into the exact nature of lepra bubalorum, a condition which has so far only been described in Indonesia, is relevant.

## Author Contributions

WF, HM, and TP: conceptualization. WF: data curation, investigation, methodology, and writing—original draft. TP: project administration, resources, and supervision. WF, HM, VR, and TP: writing—review and editing. All authors contributed to the article and approved the submitted version.

## Conflict of Interest

The authors declare that the research was conducted in the absence of any commercial or financial relationships that could be construed as a potential conflict of interest.

## Publisher’s Note

All claims expressed in this article are solely those of the authors and do not necessarily represent those of their affiliated organizations, or those of the publisher, the editors and the reviewers. Any product that may be evaluated in this article, or claim that may be made by its manufacturer, is not guaranteed or endorsed by the publisher.
